# Pleomorphic adenoma of the external auditory canal

**DOI:** 10.4322/acr.2023.428

**Published:** 2023-04-13

**Authors:** Sushma Aradhya, Namratha Ravishankar, Suchitha Satish

**Affiliations:** 1 JSS Medical College and Hospital, JSS Academy of Higher Education and Research, Department of Pathology, Mysore, Karnataka, India.

**Keywords:** Ear Canal, Immunohistochemistry, Pathology, Surgical

## Abstract

Pleomorphic adenoma (PA) of the external auditory canal (EAC) is a rare clinical entity with a few cases reported in the literature. The clinical diagnosis of these lesions can be formidable due to their rarity and unusual location. This tumor occurs at various other anatomical locations apart from the major salivary glands. A 30-year-old female presented with a two-year history of a gradually enlarging and painless mass in the left external auditory canal. The tumor was excised, and histopathological and immunohistochemical evaluation revealed a mixed tumor with both epithelial and stromal components of different proportions, recognized and classified today by the World Health Organization (WHO) as a pleomorphic adenoma. The post-operative course was uneventful, and at the 10-month follow-up, no recurrence of the pleomorphic adenoma was noted. We highlight the histological features and the immunohistochemical profile of the tumor and review the literature on glandular neoplasms of the EAC and their recent classification, emphasizing on the histogenesis, clinical presentations, and microscopic features of the tumor. In addition, we aim to discuss vital features in differentiating these tumors from other tumors of the external auditory canal to enable clinicians and pathologists to recognize this uncommon benign neoplasm.

## INTRODUCTION

Pleomorphic adenoma constitutes about 80% of benign salivary gland tumors and is characterized by slow growth and an indolent course. This tumor has been reported at various other anatomical sites apart from the major salivary glands, with one of the rare sites being the external auditory canal (EAC). Other atypical sites include the nasal septum, tongue, turbinate, upper lip, lungs, trachea, and lacrimal glands.^1^

Glandular tumors of the EAC are extremely rare, with less than 150 cases reported worldwide. The nomenclature, classification, tissue of origin, and accurate diagnosis of these tumors are still controversial. The origin of the mixed tumor of the skin or pleomorphic adenoma in this site is hypothesized to be the apocrine duct of the follicular-sebaceous-apocrine unit localized deeply in the skin lining of the external auditory canal.^2-4^ EAC tumor is usually seen in patients between 20 and 60 years as a slowly growing, encapsulated, painless, solitary, subcutaneous mass within the dermis or subcutaneous fat.^5^ The long-term clinical outcome is excellent following complete surgical excision.^6^

However, a major challenge for the surgeon and the pathologist is arriving at the diagnosis among a relatively large number of benign and malignant lesions of the EAC. This case is reported for its rarity and to discuss this tumor’s diagnosis and differential diagnoses along with the relevant literature.

## CASE REPORT

A 30-year-old female presented with a painless mass in the left external auditory canal. History was negative for hearing impairment, tinnitus, trauma, or previous surgery. The lesion was present over the last 2 years and had gradually increased. There was no associated pain, and no skin changes were noted. Otoscopy revealed a smooth, non-tender lesion covered by normal skin, almost obstructing the external auditory meatus.

The tympanic membrane could not be visualized due to the mass. There was no parotid swelling, and cervical lymph nodes were not palpable.

On palpation, the mass was tense and firm, with intact overlying skin. A computed tomography (CT) scan of both temporal bones was carried out, which was inconclusive except obliteration of the outer end of the left EAM. The tympanic cavity and mastoid cleft were normal, and no intracranial involvement was seen.

**Figure 1:**
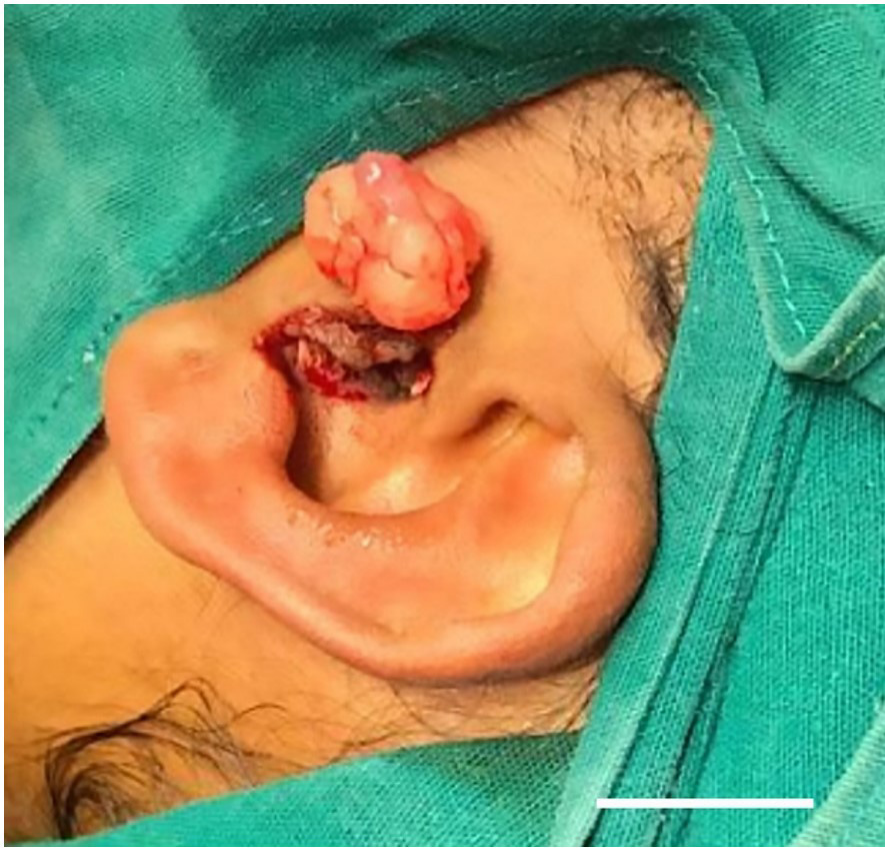
Per operatory image of the tumor in the left external auditory canal (scale bar= 2cm).

The mass was removed in-toto with overlying skin and 3 mm skin margins all around through the endaural approach (Figure 1), and the excised tumor mass was sent for histopathological examination.

On gross examination, the mass was a single, well-defined, globular, firm, grey-white tissue mass measuring 1.7x1.5x1cms. The cut section was homogenous and grey white with slit-like spaces.

**Figure 2:**
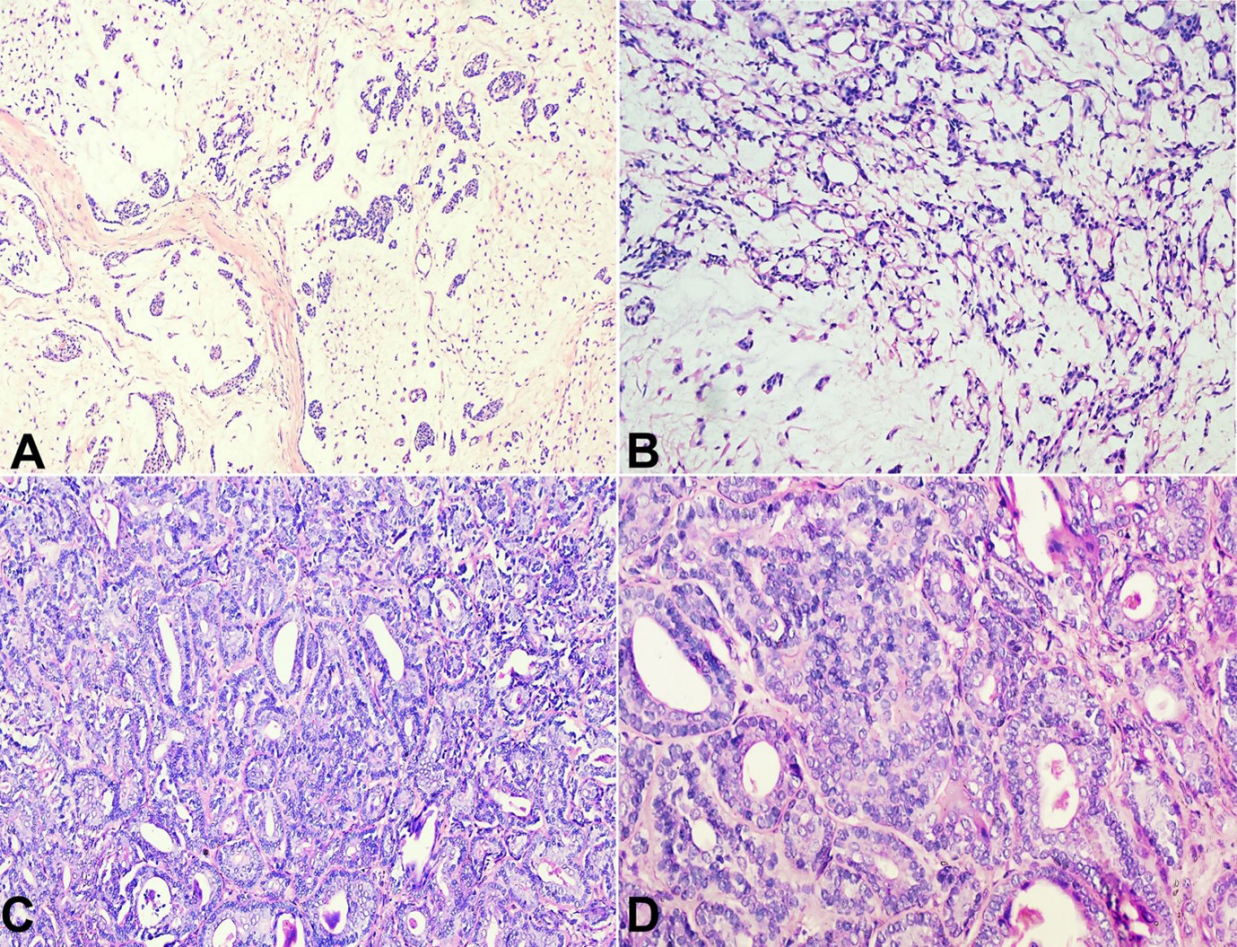
Photomicrographs of the pleomorphic adenoma. **A** and **B** show tubules, ducts, and cysts embedded in a chondromyxoid stroma. (H&E, 40X); **C** and **D -** Photomicrographs of cellular foci in the pleomorphic adenoma showing tubules lined by bilayered epithelium. (H&E, 40X and 100X respectively).

Histopathological examination revealed a well-circumscribed tumor with a lobulated growth pattern. composed of epithelial and stromal components. The epithelial component is formed by tubules, nests and acinar structures admixed with myoepithelial cells and is set in a myxoid stroma. (Figures 2A and 2B).

**Figure 3:**
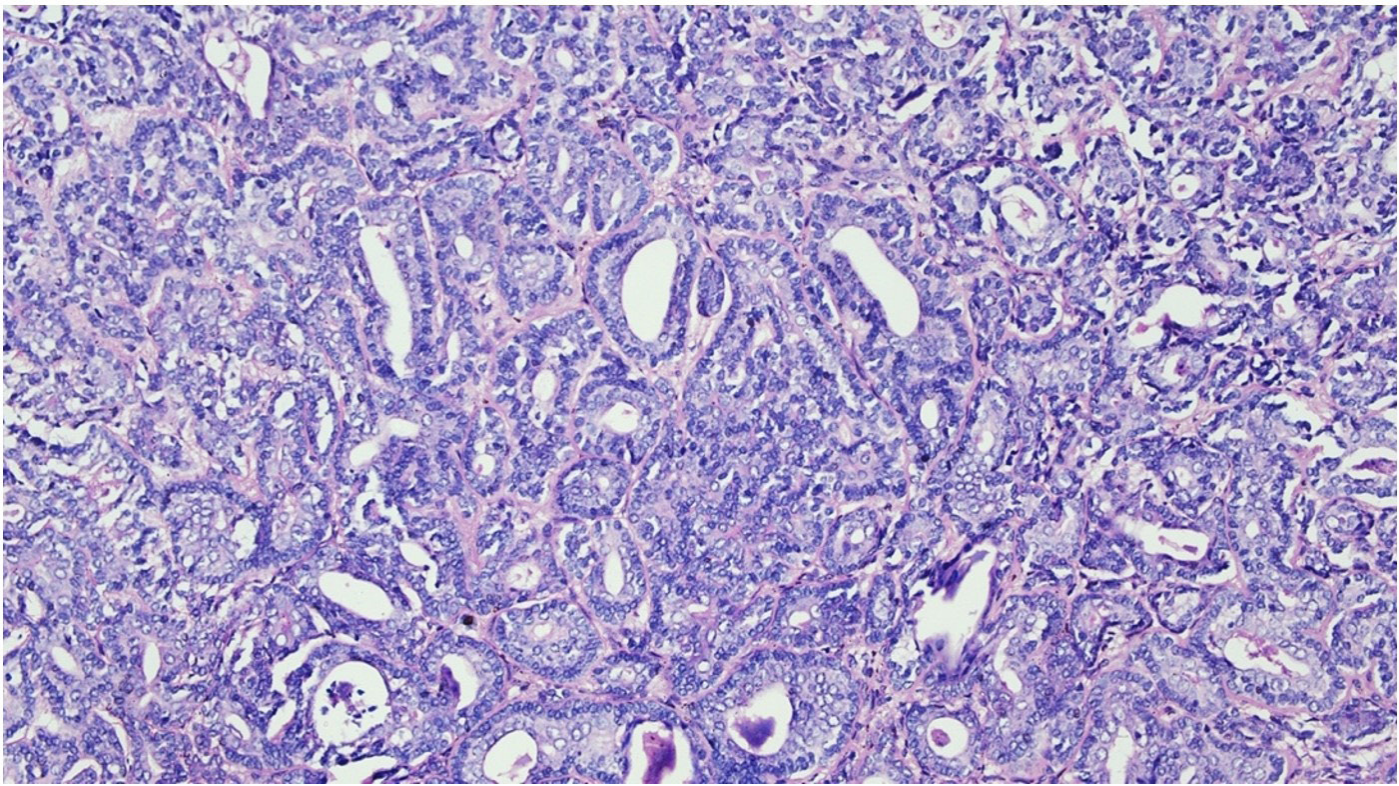
High power microscopy of a cellular area of the pleomorphic adenoma showing tubules and acinar structures lined by bilayered epithelium. (H&E, 200X).

The aforementioned tubules and acini were lined by two-layered epithelium, outer cuboidal and inner columnar epithelium. There was no nuclear pleomorphism, increased mitosis or perineural invasion. (Figure 3)

**Figure 4:**
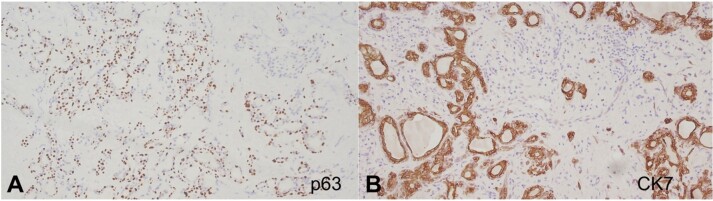
Photomicrograph of the tumor. **A -** Immunohistochemistry reaction for p63 showing positivity in the myoepithelial cells, but negative in glandular epithelial cells (200X); **B -** Immunohistochemistry reaction for CK7 highlighting the luminal layer while the abluminal myoepithelial cells are negative (200X).

A diagnosis of pleomorphic adenoma was made with histopathology. Immunohistochemistry confirmed the presence of two distinct cell populations. The luminal cells expressed cytokeratin 7 (Figure 4A), while peripheral (basal) cells expressed p63 (Figure 4B).

The postoperative course was uneventful, and the excision site was well healed with no recurrence during a 10-month follow-up.

## DISCUSSION

Primary glandular neoplasms of the external ear canal are rare and constitute only 5.7% of all neoplasms of the ear (pinna) and EAC.^7^

The accepted theory is that these glandular tumors arise from ceruminous glands, which are modified sweat glands of the skin of the external auditory meatus.^8^

These specialized glands are no longer considered purely apocrine glands, but apo-eccrine glands with both apocrine and eccrine modes of secretion. The histologic origins of glandular tumors of the EAC have numerous conflicts. Some authors have even speculated that these tumors may arise from the ectopic salivary tissue in the external ear canal.^9,10^

The apo-eccrine glands are coiled tubular glands akin to eccrine glands of the skin, whose ducts empty their contents into a hair follicular infundibulum or directly onto the skin surface. Histologically, they are characterized by clusters of tubules lined by two concentric layers of the epithelium. The prominent inner or luminal layer shows large cuboidal to columnar cells with abundant eosinophilic cytoplasm and oval nuclei located at the basal aspect of the cells. The cells may contain brownish-yellow lipofuscin/ceroid pigment along the luminal aspect of the cells, which is positive for periodic acid-Schiff, Sudan black, and acid-fast stains.^11^

Tumors arising from the ceruminous glands were first described in 1894 by Haug^12^ and were previously all referred to as ceruminoma in the literature, irrespective of their type.^13^

In 1991, the term ‘ceruminoma’ was suspended as it implied a more general definition comprising a heterogenous group of external ear canal tumors with various neoplastic potentials.^13,14^

Unilateral conductive hearing loss is the most common symptom of the pleomorphic adenoma of the external auditory meatus. Occasionally pain and otorrhea can result from otitis externa secondary to meatus obstruction.^15^ The largest series of ceruminous adenomas to date reported an average symptom duration of 16 months.^16^

Cankar and Crowley^17^ presented the first systematic classification of tumors arising in the ceruminous glands, followed by Wetli et al.^18^ into the following groups: (1) Ceruminous adenoma, (2) Pleomorphic adenoma, (3) Ceruminous adenocarcinoma, and (4) Adenoid-cystic carcinoma. In due time, this classification was expanded by Mansour et al.,^10^ as recommended presently by the WHO.^10,19^

The latest WHO classification categorizes benign tumors of ceruminous glands as adenoma NOS, pleomorphic adenoma, and syringocystoma papilliferum (Table 1).

**Table 1:** Ceruminous neoplasms of the External auditory canal according to the WHO classification

Ceruminous gland tumors		
Benign	Malignant	
Ceruminous adenoma		Ceruminous adenocarcinoma
Ceruminous pleomorphic adenoma		Ceruminous adenoid cystic carcinoma
Ceruminous syringocystadenoma papilliferum		Ceruminous mucoepidermoid carcinoma

In 1951, Mark and Rothberg^20^ reported the first case of pleomorphic adenoma arising in the external auditory canal. Pleomorphic adenomas of the EAC display histopathology akin to the tumor at other sites with ceruminous differentiation features, such as yellow ceroid pigment and decapitation secretion. Architectural patterns may be solid, including back-to-back glands and papillary with a dual cell population of inner/luminal epithelial cells and outer basal/myoepithelial cells. Cellularity is usually low or moderate, with mild nuclear pleomorphism. Nuclei are round to oval with fine, granular chromatin and small nucleoli. Luminal cells are columnar to cuboidal, with well-defined cell borders and abundant eosinophilic cytoplasm, usually showing apical caps and decapitation secretion. Most tumors contain cells with cytoplasmic golden-yellow/brown (ceroid) pigment granules.^21^

Ceruminous gland adenomas (CGA) are the most common benign glandular neoplasms of the EAC.^7^ CGAs are diagnosed typically in adults, on average, in the 6th decade of life. However, rare cases of pediatric CGA have been described, arising de novo^16,22^ or in association with nevus sebaceous.^14^

CGAs are non-encapsulated, well-circumscribed tumors composed of glandular structures lined by two layers of epithelium, typically arranged in lobulated clusters. Focal papillary structures, solid and cystic patterns of growth may be observed, and the luminal cells contain abundant eosinophilic cytoplasm with scattered yellowish-brown cytoplasmic pigment, while the myoepithelial cells may be variably prominent.^23^

The ceruminous syringocystadenoma papilliferum is the rarest benign tumor of the ceruminous glands.^7^ It is characterized by multiple short, thick papillae lined by two-layered epithelium projecting into cystic spaces with plasmacytic infiltrates in the fibrovascular cores.^23^

Malignant ceruminous neoplasms also called ‘ceruminous adenocarcinomas’ in the WHO classification of head and neck tumors, appear slightly more common than their benign counterparts. However, whether this is due to reporting bias is unclear. They include adenoid cystic carcinoma, adenocarcinoma, not otherwise specified, and mucoepidermoid carcinoma in the descending order of frequency.^7,19^ Another rarely reported malignancy of ceruminous gland origin is mucinous carcinoma.^24^

The term ‘’chondroid syringoma” is used to describe a pleomorphic adenoma arising from skin appendages, but authors have opined that the term “chondroid syringoma” can be used interchangeably with pleomorphic adenoma in the EAC.^25,26^

Tumors of the EAC are usually small and localized, and early diagnosis is extremely challenging. Due to the complex anatomy of this region, assessing the extent of tumors clinically is cumbersome, and obtaining an adequate biopsy is also challenging, leading to superficial, fragmented or partial biopsies in many cases.^27^

Due to the rarity of ceruminous neoplasms, they are frequently a diagnosis of exclusion. Benign and malignant neoplasms from ceruminous glands have overlapping clinical features and morphologic characteristics. Therefore, other tumors of epidermal, EAC, middle ear, and mastoid origins, such as cylindroma, paraganglioma, basal cell carcinoma, and neuroendocrine tumors of the middle ear, constitute the major differential diagnoses.^23^ Complete histologic and immunohistochemical evaluation are essential for accurate diagnosis.

Table 2 summarizes the common differential diagnoses’ histologic features and relevant immunohistochemical studies.

**Table 2:** Histologic features and immunohistochemical studies of the common differential diagnoses of pleomorphic adenoma of the external auditory canal

**Tumor**	**Histopathology**	**IHC**
Ceruminous gland adenoma	Glandular structures lined by two-layers of epithelium typically arranged in lobulated clusters with focal papillary structures, solid and cystic growth patterns. The stroma may be hyalinized.	The luminal cells: Cytokeratin 7. Peripheral (basal) cells: Keratins 5/6, S100 protein, and p63. Apocrine gland-related antigen GCDFP-15 focally expressed by tumor cells.
Eccrine cylindroma	Circumscribed tumor composed of a lesion limited to EAC with solid nests of luminal and myoepithelial cells and hyaline material surrounded by a hyaline sheath	Luminal cells: CEA, CK7, CK19, EMA. Myoepithelial cells: S100, p63, SMA, CK5/6
Basal cell carcinoma	Nests and infiltrative cords of basaloid cells with minimal clear cytoplasm, with peritumoral clefting and variably myxoid stroma	p63, p40, CK5/6, Ber-EP4, CD10 (periphery of tumor nests)
Neuroendocrine tumor	Cribriform, trabecular, nested, lobulated or solid proliferation of small to medium-sized cells with finely speckled chromatin surrounded by fibrotic stroma	CK7, CK5/6, p63, synaptophysin, chromogranin A, CD56
Paraganglioma	Organoid or nested growth of epithelioid (chief) cells with eosinophilic cytoplasm, ovoid nuclei and speckled chromatin, and inconspicuous spindled (sustentacular) cells	Chief cells: synaptophysin, chromogranin A, S100, cytokeratin. Sustentacular cells: S100, GFAP

Hicks,^28^ who first described neoplasms in the external auditory canal (EAC) of ceruminous gland origin, stated that accurate histopathologic evaluation could determine the natural course and clinical approach to these tumors. He also emphasized that early wide excisional biopsy is imperative for diagnosis and that the signs and symptoms of the tumor do not always correlate with the histopathologic diagnosis and subsequent clinical behavior.

Pre-operative assessment of the swelling in our case was not done but studies have shown that pleomorphic adenoma of the EAC can be diagnosed on fine-needle aspiration cytology (FNAC) prior to surgery, and a cytopathologist should be well aware of its cytological findings to avoid any misdiagnosis.^29^ However its ability to identify malignant features, such as increased mitoses, may be limited by sampling.^30^

The mean age for the presentation of pleomorphic adenoma is the fifth decade; however, it may range from 12 to 85 years.^9^ Clinically, the tumor usually presents as a smooth, soft, non-tender, polypoid mass in the EAC protruding from the EAC, depending on its size. The tympanic membrane usually remains intact unless chronic inflammation coexists in the middle ear. The lesion size is variable and depends on the time between its first manifestation and diagnosis. In comparison with other benign adenomas of the EAC (mean diameter, 1.15 cm), its size is somewhat larger (mean diameter, 1.4 cm)^7^ The commonest site of origin of the tumor is the posterior or posterosuperior canal wall as in this case.^31^

Symptoms depend on the tumor size and the extent to which the EAC is occluded. Noteworthy symptoms include conductive hearing loss, a sensation of fullness in the ear, otalgia, tinnitus, hemorrhage, and otorrhea.^7,31^

Macroscopically these tumors are circumscribed without being encapsulated.

When removed intact, they appear as polypoid masses covered by a non-ulcerated epithelium. To date, the largest case series of these tumors reports a mean size of 1.1 cm and a range of 0.4-2.0 cm.^7^ Rarely cystic change may be seen.^6^

The diagnosis of PA is usually made retrospectively based on histopathological findings consisting of tubuloductal epithelial structures, glands, and small cysts lined by a tubuloglandular proliferation of cerumen-secreting cells with decapitation secretion surrounded by a spindled to cuboidal myoepithelial layer set in a chondroid matrix.^5-7^ The stromal component is usually chondromyxoid but other stromal components such as fibrous, fatty, hyalinized, osteoid have been described.^32^

Lipomatous pleomorphic adenoma arising from the ceruminous glands with extensive mature adipocytes and spindle-shaped myoepithelial cells set in a fibromyxoid stroma has also been described.^33^

Cerumen pigment, CK7, and p63 can help to differentiate this tumor from other neoplasms in the region.^7^ Many luminal cells may show membranous expression of CD117/c-Kit, and the apocrine gland-related antigen GCDFP-15 may be focally expressed by tumor cells.^34^ The tumor cells may also be positive for Glut-1, HIF-1α, PI3K and p-Akt.^35^ The stromal cells show myoepithelial differentiation and are immunoreactive for p63, S100, vimentin, neuron-specific enolase(NSE), glial fibrillary acid protein(GFAP) and smooth muscle actin(SMA).^32^

Similar to the pleomorphic adenoma of the salivary glands, relapses may be seen in the EAC if resection is inadequate or if the tumor ruptures during surgery and a case of local relapse, six years post resection, has been described.^36,37^

Although the malignant transformation of these tumors is rare, there are reported cases of pleomorphic adenomas of the EAC progressing to aggressive forms. This mandates adequate surgery and long-term follow-up of these tumors owing to their potential to recur and progress to malignancy^5,38^

Malignant relapse of pleomorphic adenoma of the EAC with satellite nodules, cellular atypia, and mitotic activity and metastasis has been described,^30,38^ and a rare case of epithelial-myoepithelial carcinoma from a pleomorphic adenoma in the EAC have also been chronicled.^39^

In malignant transformation of PA, common locations of metastases are regional lymph nodes, lungs, and bone. Histologically, these are recognized by cellular atypia, increased mitotic activity, infiltrative margins, satellite tumor nodules, and tumor necrosis.

Sufficient surgical therapy with wide margins is hence advocated, accompanied by regular and long-term follow-up as recurrence may occur after complete local excision.^25,30^

Malignant tumors of the EAC mandate early aggressive surgery and radiotherapy. When marginal invasion cannot be assessed by microscopy, it is suggested by authors that the tumor be reported as 'of uncertain malignant potential'. Long-term, extensive studies are imperative to verify or disprove the theory that all ceruminous gland tumors possess malignant potential.^10^

## CONCLUSION

Ceruminous neoplasms are uncommon tumors of the external auditory canal that present with protean clinical presentations with considerable morphologic overlap between benign and malignant neoplasms. The rarity of the pleomorphic adenoma in the EAC renders the identification difficult. Definite diagnosis may only be reached with histological and immunohistochemical analysis of the biopsy or the excised tumor specimen. Due to the risk of recurrence and malignant transformation, treatment of seemingly benign neoplasms of the EAC should comprise excisional biopsy with adequate safety margins along with long-term follow-up. Although pleomorphic adenoma is extremely rare, it should always be considered in the differential diagnosis of a mass in the EAC.
